# The Causation and Treatment of Scoliosis

**Published:** 1909-07-03

**Authors:** 


### Surgery.

### THE CAUSATION AND TREATMENT OF SCOLIOSIS.

There are many causes of scoliosis, but the variety which is most commonly met with is that which comes on in early adolescence, generally in young girls, and it is also, fortunately, the variety in which efficient treatment, if patiently persisted in, is likely to give the best result. The patient herself is, as often as not, quite unconscious that anything is wrong; it is usually her relations or friends who bring her to seek advice because they have noticed what they describe as " a growing out of the right shoulder."

### Ætiological Factors.

The factors producing this are the same as in all deformities which come on in adolescence. This period is associated with undue growth, and the muscular system does not develop at the same rate as the bony skeleton, and it therefore becomes functionally inefficient. In cases of scoliosis the erector-spinæ group is the one affected. But why should this cause " a growing out of the right shoulder "? The reason is this: The vertebral column is not absolutely straight even in a perfectly normal individual. There is a slight curve of the dorsal spine, with its convexity towards the right. Two explanations have been put forward to account for this: (1) that the object of the curve is to allow room for the heart and aorta in the left side of the thorax, and (2) that in right-handed people (and these form so large a majority that they must be regarded as normal individuals) the greater use of the right arm strengthens the muscles which attach it to the trunk, so that they gradually pull the vertebral column out of the straight. The first explanation seems the more feasible of the two.

As soon as the muscles become insufficient to support the vertebral column, the normal curve to the right becomes more and more marked. Two secondary curves, both of which are convex to the left, occur later, one in the cervical and the other in the lumbar region. These are the result of the patient's desire to carry the head and body erect.

But these are not the only anatomical changes found. The vertebræ revolve on their vertical axis, so that the spine in each case turns towards the concavity of the curve. In the thorax the vertebræ as they turn carry the ribs and sternum round with them, so that the sternum is displaced to the right, but it tends to return to the middle line. This is achieved by a change in the shape of the entire thorax, and two prominent acute angles are formed, one on the left side in the anterior axillary line, and the other on the right side in the posterior axillary line. The anterior prominence so caused may be most misleading. If it is more marked on one rib than in the rest, it may be mistaken for an exostosis; or if, as often happens, the second and third ribs are most affected, the outer half of the breast may appear to be unusually protuberant, and this organ may be thought to be at fault.

In examining a case of scoliosis, the first thing to do is to mark out carefully with a flesh pencil the position of all the vertebral spines. The degree of curvature thus shown is less than the real one present because, as has been already mentioned, the vertebræ rotate so that their spines lie towards the concavities. The patient should then be made to hang by her hands from a horizontal bar, and a second marking of the spines taken in this position. It is, of course, more accurate to take two skiagraphs, one in the supine and the other in the hanging position, but this is not always practicable.

Cases of scoliosis may be divided into two classes according to the results of this examination: (*a*) those in which the curvature is obliterated in the hanging position, and (*b*) those in which little or no change is seen. In the first a good prognosis may be given as to the ultimate result provided that treatment is patiently persisted in, but in the second it is to be presumed that the ligaments on the concave aspects of the curves have been secondarily shortened, and in this case treatment will not effect a cure. At the best it will only arrest the progress of the disease.

### Methods of Treatment.

The treatment consists of exercises which can be carried out at home by the patient. The most useful are the following: (1) hanging on a horizontal bar and pulling the body up until the chin is level with the hands, (2) stooping down and making the tips of the fingers touch the toes without bending the knees, and (3) alternately raising and lowering the body while resting on the hands and toes. These exercises must be graduated; that is to say, the patient must begin by doing them only a few times, it being most important that *she should never be allowed to fatigue herself.* The number of times that any exercise can be done without getting tired is soon learned by experience. At first she should do the exercises twice a day, and after each occasion she should be kept absolutely at rest, flat on her back, for two hours.

Massage to the back is also extremely beneficial, and if this treatment is given it should be applied for half an hour before each period of rest. It will be found, as time goes on, that the patient can undertake a progressively greater number of exercises without fatigue. The back should be examined from time to time in order that it may be known how much benefit is being derived from the treatment. As soon as the back is straight without hanging the patient up the treatment may be allowed to become a little less rigorous, but the habit of doing a few exercises daily should be kept up until adult life is reached.

A poroplastic jacket usually does more harm than good, since it gives the weakened muscles an artificial support, on which they learn to rely, instead of being encouraged to do their own work efficiently.

